# Exogenous Nucleotides Ameliorate Age-Related Decline in Testosterone in Male Senescence-Accelerated Mouse Prone-8 (SAMP8) Mice by Modulating the Local Renin–Angiotensin System Antioxidant Pathway

**DOI:** 10.3390/nu15245130

**Published:** 2023-12-17

**Authors:** Qianqian Chen, Rui Liu, Chan Wei, Xiujuan Wang, Xin Wu, Rui Fan, Xiaochen Yu, Zhen Li, Ruixue Mao, Jiani Hu, Na Zhu, Xinran Liu, Yong Li, Meihong Xu

**Affiliations:** 1 Department of Nutrition and Food Hygiene, School of Public Health, Peking University, Beijing 100191, China; 2 Beijing Key Laboratory of Toxicological Research and Risk Assessment for Food Safety, Peking University, Beijing 100191, China

**Keywords:** exogenous nucleotides, testosterone, renin–angiotensin system, oxidative stress

## Abstract

In older men, an age-related decline in testosterone is closely associated with various adverse health outcomes. With the progression of aging, hyperactivation of the local renin–angiotensin system (RAS) and oxidative stress increase in the testis. The regulation of RAS antioxidants may be a target to delay testicular aging and maintain testosterone levels. Exogenous nucleotides (NTs) have anti-aging potential in several systems, but there are no studies of their effects on the reproductive system. In our study, we examined the effects of exogenous NTs on testosterone synthesis and explored possible mechanisms of action. Therefore, senescence-accelerated mouse prone-8 (SAMP8) mice and senescence-accelerated mouse resistant 1 (SAMR1) were used in the experiment, and they were randomly divided into an NTs free group (NTs-F), a normal control group (control), a low-dose NTs group (NTs-L), a middle-dose NTs (NTs-M), a high-dose NTs group (NTs-H) and SAMR1 groups, and the testis of the mice were collected for testing after 9 months of intervention. The results showed that exogenous NTs could increase the testicular organ index in mice during aging, and delayed the age-associated decline in testosterone levels in SAMP8 male mice, possibly by modulating the local RAS antioxidant pathway and reducing oxidative stress to protect the testis. The present study provides new research clues for the development of preventive and therapeutic strategies for related diseases.

## 1. Introduction

An age-related decline in testosterone is defined as a gradual decrease in body testosterone with increasing age in men, which is most pronounced in the seventh decade of life [1,2]. In the older male population, nearly 23.3% of those experiencing reduced testosterone levels develop late-onset hypogonadism [3], which is characterized by sexual dysfunction, reduced bone density, decreased muscle mass, cognitive impairment, and mental disorders. In addition, it is associated with an increased risk of diabetes and all-cause and cardiovascular mortality [4,5]. Thus, it can seriously affect the physical and mental health and quality of life of older adults [6,7]. Also, there is clear evidence that older male offspring are at an increased risk of specific genetic diseases associated with their father’s age [8]. The prevalence of the disease increases every year, following along with the aging process. Therefore, age-related declines in testosterone not only affect reproductive function and genetics, but have more significant public health implications for older populations and for society. However, current studies in anti-reproductive aging seem to favor anti-ovarian aging, while studies in anti-testicular aging are less common. Currently, testosterone replacement therapy (TRT) is most widely used in the clinical management of testosterone deficiency. However, the long-term safety of TRT has not been clarified [7,9].

The renin–angiotensin system (RAS), which acts mainly through renin, angiotensin I (Ang I), angiotensin II (Ang II), angiotensin-converting enzyme (ACE) and aldosterone (ALD), is recognized as a circulating hormone system [10,11]. However, in recent years, it has been shown that the main family members of the RAS are localized to testicular tissues [10,12,13]. Moreover, their transcripts were found to be synthesized in Leydig cells [12,14,15], suggesting that the local RAS regulates testosterone production. Ang II, as the main active substance of the RAS, induces oxidative stress and reduces testosterone production [16,17,18,19]. ACE2 acts as a constraint on the effects of the RAS and exerts a physiological role in the fight against oxidative stress [20]. ACE2 is selectively expressed in Leydig cells in the testis and promotes testosterone production [21,22]. In addition, some studies have reported that renin levels are directly correlated with testicular testosterone concentrations [23]. Also, as a steroid hormone, ALD may affect testosterone production [24].

Increased oxidative stress is a major feature of aging and is associated with a variety of age-related diseases. Nrf2, a key transcription factor in the regulation of anti-oxidative stress, plays an important role in inducing an antioxidant response in the organism [25]. Notably, Nrf2 plays a critical role in preventing male sterility, as Nrf2-null male mice develop sterility in an age-dependent manner [26]. It has been shown that overactivation of the RAS enhances oxidative stress in testicular tissue, accelerates apoptosis and leads to reduced testosterone production [27]. Thus, regulation of the RAS, the activation of Nrf2 and the enhancement of antioxidant capacity may be an important target for intervening in age-associated declines in testosterone levels. In addition, it has been shown that Leydig cells secrete testosterone according to biological rhythm, as the biological clock gene Bmal1 regulates the expression of genes related to the synthesis and secretion of testosterone [28], and it can regulate the expression of Nrf2 by binding to E-box [29]. Additionally, the expression of some of the major RAS members is similarly regulated by circadian rhythms [30].

Nucleotides (NTs) are regulators of biological processes in the body and are important for growth, development, metabolism, reproduction and genetics, and can be synthesized by the body itself [31]. Exogenous NTs are considered to be conditionally essential nutrients. Under normal physiological conditions, NTs produced in the body and consumed in the daily diet can meet the needs of the organism. However, during aging, the above pathways are not sufficient to meet the body’s needs, and supplementation with exogenous NTs is considered necessary. Long-term deficiencies in the intake of NTs may lead to multi-system dysfunction. Several studies have shown that exogenous NTs have antioxidant [32,33], immunity [34], intestinal flora [35] and memory [36] effects, with the potential to delay aging. Oxidative stress is a major feature of aging, so as natural antioxidants, exogenous NTs may have potential anti-aging effects on the reproductive system. However, the effects of exogenous NTs on the reproductive system have not been reported.

Therefore, the aim of the present study was to examine the effects of exogenous NTs on testosterone levels in male SAMP8 mice, to investigate whether NTs contribute to the modulation of the local RAS antioxidant capacity, and to provide a safer prevention and treatment strategy against age-related testosterone decline in aging men, from the perspective of nutritional intervention.

## 2. Materials and Methods

### 2.1. Test Substances

A mixture of exogenous NTs (5′-AMP:5′-CMP:5′-GMPNa_2_:5′-UMPNa_2_ = 16:41:19:24) was produced by yeast enzymatic production and isolation with a purity of >99%, supplied by Zhen-Ao Biotechnology Co., Ltd. (Dalian, China).

The standard feed was the American Institute of Nutrition Rodent Diets-93M (AIN-93M); the purified feed containing free NTs was extracted from the standard feed, and the NT intervention feeds were produced by adding different doses of NTs to standard feeds. The above customized feeds for each group were provided by Beijing KAO Co-operative Feeding Co Ltd. (Beijing, China).

### 2.2. Animals and Treatment

Male 12-week-old specific-pathogen free (SPF) SAMP8 and SAMR1 mice were provided by the Laboratory Animal Center of Peking University. They were housed in barrier-grade animal rooms in single cages, at 24 ± 2 °C, with a relative humidity of 50–60% and 12 h alternating lighting. During the experimental period, the animals were free to eat and drink.

After 1 week of adaptation feeding, 75 SAMP8 mice were randomly randomized into five groups, with 15 mice in each group: NTs free group (NTs-F), normal control group (control), low-dose NTs group (NTs-L), middle-dose NTs (NTs-M) group, and a high-dose NTs group (NTs-H). Meanwhile, 15 SAMR1 mice were set up as the SAMR1 model control group (SAMR1). The control and SAMR1 groups were given standard feed, and NTs-F group was given purified feed. For the NT intervention groups, NTs were added to the standard feed at different doses of 0.3, 0.6 and 1.2 g/kg. Animal experiments and intervention groups are shown in Table 1. The food intake and the body mass of the mice were measured and recorded weekly. Mice were executed at 12 months of age and the execution time was standardized to the morning with 8 h of fasting before execution. Bilateral testes were removed and weighed, and immediately stored at −80 °C for further examination.

### 2.3. Testicular Organ Index

Mouse body weights were measured weekly, and then the bilateral testicular tissues of the mice were removed, rinsed with saline, and weighed to calculate the organ coefficient, using the following formula: testicular organ index = bilateral testicular mass (g)/body weight (g) × 100%.

### 2.4. Testing of Testosterone in Testis

A 10% testicular tissue homogenate supernatant was prepared by taking 60 mg of testicular tissue and adding 600 μL of 0.9% normal saline, which was later used for the test kit assay. According to the kit instructions, the testosterone levels in testicular tissue were measured using radioimmunoassay. The testosterone assay kit was purchased from BNIBT, Beijing, China.

### 2.5. Testing of the Main Renin–Angiotensin System Molecules in Testes

Radioimmunoassay was used to measure the levels of Ang II (BNIBT, Beijing, China) and ALD (BNIBT, Beijing, China) in testicular tissue. ELISA was used to measure the levels of ACE2 (BG, Shanghai, China), renin (Boster, Wuhan, China), renin receptor (PRR, Abclonal, Wuhan, China) and mineralocorticoid receptor (MR, Abclonal, Wuhan, China) in testicular tissue. All of the above indicators were tested according to the test kit instructions.

### 2.6. Testing of Antioxidant Pathway-Related Indicators

Three mice were randomly selected from each group for Western blot analysis, used to determine Nrf2 protein expression. Frozen tissue protein lysate was taken for sample preparation. Twenty mg of testicular tissue was taken; 200 μL of protein lysate was added for every 10 mg of tissue and homogenized on ice using a glass grinder. Next, the homogenate was transferred to pre-cooled 1.5 mL EP tubes and placed on ice for 15 min to become fully lysed, before being centrifuged at 12,000 rpm for 10 min at 4 °C. The centrifuged supernatant was transferred in portions to 0.5 mL centrifuge tubes and frozen at −20 °C for further analysis. The total protein concentration was determined by following the instructions of the BCA Protein Quantification Kit (Applygen, Beijing, China). The lysates were separated using gel electrophoresis and then transferred to a PVDF membrane. Five g of skim milk powder was dissolved in 100 mL of TBST to prepare 5% skim milk powder, which was used as a blocking agent. The membrane was immersed and kept at room temperature for 4 h. After blocking, the membrane was incubated with a primary antibody at 4 °C overnight. Then, the membrane was incubated with secondary antibody at 4 °C for 4 h. Beta-actin (42 KDa) was used as an internal reference protein. NRF2 (D1Z9C) XP Rabbit mAb #12721 was purchased from CST, Danvers, MA, USA. Anti-beta actin antibody ab8227 and Goat Anti-Rabbit IgG ab6721 were purchased from abcam, Cambridge, UK. Grayscale analysis was performed using Image-Pro Plus (Media Cybernetics Corp, Rockville, MD, USA).

Malondialdehyde (MDA), total superoxide dismutase (SOD) and glutathione peroxidase (GSH-Px) were determined by colorimetric assay, and the kits were purchased from Nanjing Jiancheng.

### 2.7. Testing of Bmal1 Protein Expression

Bmal1 protein expression was tested by Western blot analysis. The assay procedure was the same as Nrf2 protein expression. Anti-BMAL1 antibody ab235577, Anti-beta actin antibody ab8227 and Goat Anti-Rabbit IgG ab6721 were purchased from abcam, UK.

### 2.8. Statistics Analysis

All experimental data were displayed as mean ± SD. Data were analyzed by ANOVA using SPSS 24.0 software (IBM Corp., Armonk, NY, USA). If the variances were equal, least significant difference (LSD) was used for between-group comparisons, otherwise Tamhane’s test was used. Origin 2022 plotting was used and *p* < 0.05 was considered statistically significant.

## 3. Results

### 3.1. Exogenous Nucleotides Improve Testicular Organ Index in Aging SAMP8 Mice

**Figure 1:**
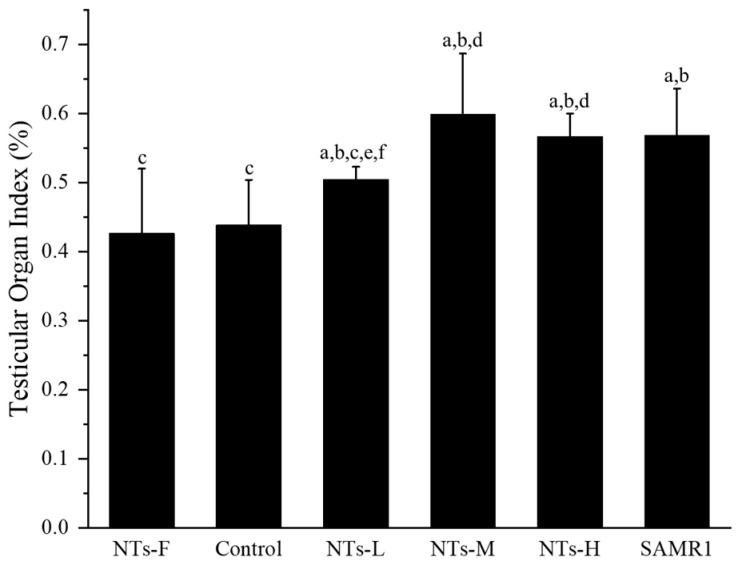
Effect of NTs on testicular organ index in aging SAMP8 mice: ^a^ compared with the NTs-F group (*p* < 0.05); ^b^ compared with the control group (*p* < 0.05); ^c^ compared with the SAMR1 group (*p* < 0.05). Among the three NT intervention groups: ^d^ compared with the NTs-L group (*p* < 0.05); ^e^ compared with the NTs-M group; ^f^ compared with the NTs-H group (*p* < 0.05).

The testicular organ index was used to assess the degree of testicular atrophy. As shown in Figure 1, the testicular organ index was higher in the NTs-L, NTs-M, NTs-H, and SAMR1 groups, compared with the control and NTs-F groups (*p* < 0.05). The NTs-M, NTs-H, and SAMR1 groups were higher than the NTs-L group (*p* < 0.05). Among the NT intervention groups, the NTs-M and NTs-H groups were higher than the NTs-L group (*p* < 0.05). Thus, NT supplementation improved the testicular organ index in aging SAMP8 mice.

### 3.2. Exogenous Nucleotides Increase Testosterone Levels in Testicular Tissue

**Figure 2:**
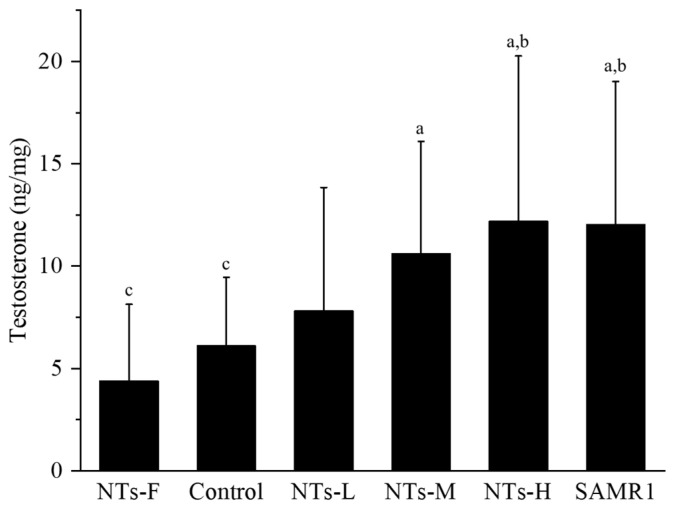
Effect of NTs on testosterone content in testicular tissue. We randomly chose 10 mice to be tested. ^a^ Compared with the NTs-F group (*p* < 0.05); ^b^ compared with the control group (*p* < 0.05); ^c^ compared with the SAMR1 group (*p* < 0.05). Among the three NT intervention groups: ^d^ compared with the NTs-L group (*p* < 0.05); ^e^ compared with the NTs-M group; ^f^ compared with the NTs-H group (*p* < 0.05).

The effects of exogenous NTs on testosterone levels in aging SAMP8 mice were detected by ELISA. As shown in Figure 2, the testosterone levels in the NTs-F group were lower than the NTs-M, NTs-H and SAMR1 groups (*p* < 0.05). It was higher in the NTs-H and SAMR1 groups, compared to the control group (*p* < 0.05), and no significant differences were observed between the NTs-L, NTs-M, NTs-H and SAMR1 groups (*p* > 0.05). This shows that the testosterone levels of SAMP8 mice were significantly increased after the intervention of exogenous NTs.

### 3.3. Exogenous Nucleotides Regulate the Levels of Key Renin–Angiotensin System Molecules in Testicular Tissue

**Figure 3:**
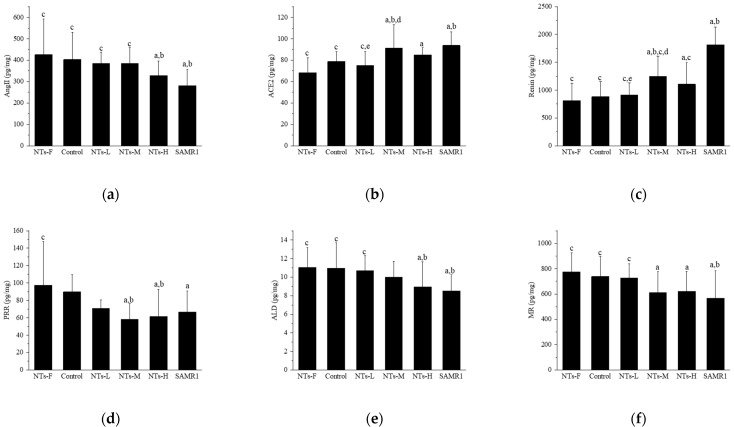
Effects of exogenous NTs on key RAS molecules in testicular tissue. We randomly chose 10 mice to be tested. (**a**) Effects of exogenous NTs on Ang II. (**b**) Effects of exogenous NTs on ACE2. (**c**) Effects of exogenous NTs on renin. (**d**) Effects of exogenous NTs on (pro)renin receptor (PRR). (**e**) Effects of exogenous NTs on ALD. (**f**) Effects of exogenous NTs on mineralocorticoid receptor (MR). ^a^ Compared with the NTs-F group (*p* < 0.05); ^b^ compared with the control group (*p* < 0.05); ^c^ compared with the SAMR1 group (*p* < 0.05). Among the three NT intervention groups: ^d^ compared with the NTs-L group (*p* < 0.05); ^e^ compared with the NTs-M group; ^f^ compared with the NTs-H group (*p* < 0.05).

To explore the mechanism of exogenous NTs improving testosterone levels in aging SAMP8 mice, we detected changes in the levels of key RAS molecules in testicular tissue after intervention (Figure 3).

In Figure 3a, Ang II levels were higher in the NTs-F group than in the NTs-H and SAMR1 groups (*p* < 0.05), and were lower in the NTs-H and SAMR1 groups than the control group (*p* < 0.05); among the three intervention groups with different doses of NTs, no intergroup differences were observed (*p* > 0.05). In Figure 3b, the ACE2 level in the NTs-F group was lower than those in the NTs-M, NTs-H, and SAMR1 groups (*p* < 0.05), was higher in the NTs-M and SAMR1 groups, compared to the control group (*p* < 0.05), and, in the intervention group, it was significantly higher in the NTs-M group than in the NTs-L group (*p* < 0.05). In Figure 3c, the renin level in the NTs-F group was lower than those in the NTs-M, NTs-H, and SAMR1 groups (*p* < 0.05), and higher in the NTs-M and SAMR1 groups than the control group (*p* < 0.05). Within the NT intervention groups, the NTs-M group was significantly higher than the NTs-L group (*p* < 0.05). In Figure 3d, for PRR levels in testicular tissue, no difference was observed between the control and SAMR1 groups (*p* > 0.05), and there was also no difference observed between the NTs-F and control groups (*p* > 0.05). In Figure 3e, ALD levels were higher in the NTs-F group than in the NTs-H and SAMR1 groups (*p* < 0.05), and were lower in the NTs-H and SAMR1 groups than the control group (*p* < 0.05). No differences were observed among intervention groups (*p* > 0.05). In Figure 3f, MR levels were higher in the NTs-F group than in the NTs-M, NTs-H, and SAMR1 groups (*p* < 0.05), lower in the SAMR1 group, compared with the control group (*p* < 0.05), and no difference was observed in the intervention groups versus the control group (*p* > 0.05).

In conclusion, the intervention of NTs at medium and high doses can affect the levels of key molecules of the RAS in testicular tissue, which can down-regulate Ang II, and up-regulate the levels of ACE2, renin, and ALD, but their effects on the levels of PRR and MR were not observed.

### 3.4. Exogenous Nucleotides Improve Antioxidant Indices and May Activate Nrf2 in Testicular Tissue

To explore the mechanism of exogenous NTs affecting oxidative stress further by regulating RAS levels and resulting in improving testosterone levels, the changes in oxidative stress-related indexes in testicular tissue were detected.

As shown in Table 2, the SOD activity in the NTs-F group was lower than those in the NTs-L, NTs-M and SAMR1 groups (*p* < 0.05), and was higher in the NTs-L, NTs-M, NTs-H and SAMR1 groups, compared with the control group (*p* < 0.05); the GSH-Px activity was lower in the NTs-F and control groups than in the NTs-L, NTs H and SAMR1 groups (*p* < 0.05). There was no difference between the NTs-L, NTs-H and SAMR1 groups (*p* > 0.05), and the NTs-H group was higher than the NTs-M group (*p* < 0.05). The MDA content was higher in the NTs-F group than in the NTs-H and SAMR1 groups (*p* < 0.05), and was lower in the NTs-L, NTs-M, NTs-H and SAMR1 groups, compared with the control group (*p* < 0.05). No differences were observed between the NT intervention groups (*p* < 0.05).

**Figure 4:**
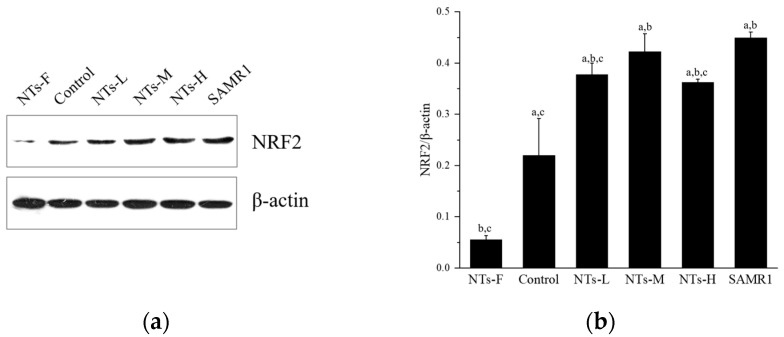
Effects of exogenous NTs on protein expression of Nrf2 in testicular tissue. We randomly chose three mice to be tested. (**a**) Immunoblots of NRF2 protein. (**b**) NRF2/β-actin. ^a^ Compared with the NTs-F group (*p* < 0.05); ^b^ compared with the control group (*p* < 0.05); ^c^ compared with the SAMR1 group (*p* < 0.05). Among the three NT intervention groups: ^d^ compared with the NTs-L group (*p* < 0.05); ^e^ compared with the NTs-M group; ^f^ compared with the NTs-H group (*p* < 0.05).

Figure 4 shows the Nrf2 protein expression in testicular tissue. Nrf2 protein expression was lower in the NTs-F group than in the control, NTs-L, NTs-M, NTs-H, and SAMR1 groups (*p* < 0.05), and was higher in the NTs-L, NTs-M, NTs-H, and SAMR1 groups than the control group (*p* < 0.05); no significant differences were observed among the NTs-L, NTs-M and NTs-H groups (*p* > 0.05). Thus, exogenous NT intervention may decrease the level of oxidative stress by affecting the level of the RAS and activating Nrf2 to increase the level of testosterone. Moreover, the long-term lack of NT intake may inhibit antioxidant pathways and increase oxidative stress levels in testicular tissue.

### 3.5. Exogenous Nucleotides May Increase Bmal1 Expression in Testicular Tissue

**Figure 5:**
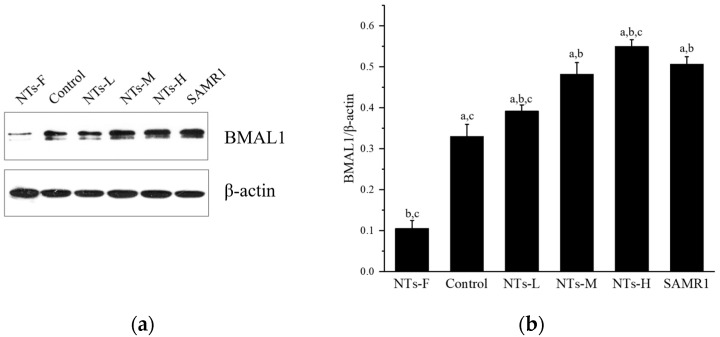
Effects of exogenous NTs on protein expression of Bmal1 in testicular tissue. We randomly chose three mice to be tested. (**a**) Immunoblots of BMAL1 protein. (**b**) BMAL1/β-actin. ^a^ Compared with the NTs-F group (*p* < 0.05); ^b^ compared with the control group (*p* < 0.05); ^c^ compared with the SAMR1 group (*p* < 0.05). Among the three NT intervention groups: ^d^ compared with the NTs-L group (*p* < 0.05); ^e^ compared with the NTs-M group; ^f^ compared with the NTs-H group (*p* < 0.05).

In addition, we explored the possible role of the biological clock-regulated gene Bmal1 in increasing testosterone levels by exogenous NTs. As shown in Figure 5, Bmal1 protein expression in the NTs-F group was lower than in the control, NTs-L, NTs-M, NTs-H, and SAMR1 groups (*p* < 0.05), and was increased in the NT intervention groups and SAMR1 groups, compared with the control group (*p* < 0.05). No significant differences were observed among the NT intervention groups (*p* > 0.05). Moreover, the NTs-L group was higher than the SAMR1 group. Thus, exogenous NTs may up-regulate Bmal1 protein expression. Also, it was shown that a prolonged deficiency of NT supplementation may down-regulate the expression of the biological clock-regulated gene Bmal1.

## 4. Discussion

This study is significant in the field of anti-aging. In this study, we used aging SAMP8 mice and SAMR1 mice to explore the effects of long-term NT intervention on testosterone production and initially explored the possible regulatory mechanisms. Currently, SAMP8 mice are widely used in aging-related studies [37,38,39]. The changes in body weight and food intake of SAMP8 and SAMR1 mice during the intervention were reported in our previous studies [33,35]. In the present study, we found that, with the progression of the aging process, the testes of mice showed significant atrophy, and the 0.6 and 1.2 g/kg NT interventions appeared to maintain the morphology of the testis. Testicular morphology is generally a simple response to male reproductive health. Testicular atrophy may indicate the presence of abnormalities or damage to the testis that can affect spermatogenesis and testosterone hormone production [40]. Testosterone is secreted by Leydig cells and promotes spermatogenesis and maturation, increases muscle mass and strength, and maintains bone density [41]. The results of the present study showed that long-term NT supplementation slowed the age-dependent decline in testosterone levels.

The presence of key molecules of the RAS has been demonstrated in the male reproductive system, mainly in the testis and epididymis. In recent years, studies have shown that the RAS can influence testosterone secretion at multiple levels and regulate male fertility [10,42,43]. In this study, changes in the levels of key RAS molecules in testicular tissue were found after long-term NT intervention, and Ang II was reported to inhibit adenylate cyclase activity in rat Leydig cells, reducing gonadotropin-stimulated cAMP and testosterone production [17]. However, ACE2 serves as a core molecule for negative feedback regulation of Ang II effects, and ACE2 cleaves Ang II to directly produce Ang (1–7), which reduces Ang II levels, and Ang (1–7) binds to Mas R to exert beneficial biological effects, such as anti-oxidative stress [10]. In the testis, Ang (1–7) is localized mainly to Leydig cells [44], and it has been shown that the activity of genes encoding steroid synthases is altered in the testes of Mas R-deficient mice [45]. Thus, ACE2 may positively regulate testosterone synthesis through the ACE2/Ang (1–7)/Mas R axis. In the present study, the results showed that Ang II decreased and ACE2 increased in testicular tissue after a 0.6 g/kg long-term NT intervention. In addition, renin/PRR and aldosterone/MR may affect testosterone production. Some studies have reported a direct correlation between renin levels and testicular testosterone concentrations [23], and a possible interaction between aldosterone synthesis and testosterone synthesis [24]. However, the synthesis of PRR and ALD/MR in the testis has not been clarified. In the present study, the results showed that PRR, ALD and MR were present in testicular tissue, and long-term NT intervention at a high dose of 1.2 g/kg appeared to increase renin and decrease ALD levels in testicular tissue, suggesting that it is possible that the modulation of renin and ALD may be stronger through systemic circulatory effects than through localized testicular tissue modulation effects.

As the aging process develops, the increased production of oxidants from various sources, along with the dysregulation of antioxidant defenses, leads to the accumulation of oxidative damage to proteins, nucleotides and lipids in senescent cells, resulting in an increase in the levels of the lipid peroxides malondialdehyde, MDA, and the protein peroxidation products, carbonylated proteins, among others [46]. Nrf2 plays a key role in the cellular counteraction to oxidative stress and the maintenance of intracellular redox homeostasis. It can activate transcription by binding to the promoter regions of anti-oxidative stress genes, increasing SOD, GSH-Px, Catalase, GST, etc. [25]. Nrf2 can be activated by a variety of bioactive substances, including natural antioxidants such as polyphenolic compounds [47,48] and thioamino acids [49]. The results of the present study suggest that long-term NT intervention may activate Nrf2 in testicular tissue, increase SOD and GSH-Px activity and decrease MDA content in testicular tissue, suggesting that exogenous supplementation with NTs may improve the antioxidant capacity of the testis. Oxidative stress is closely related to decreased testosterone secretion. It has been shown that the decline in testosterone secretion is associated with the Nrf2/ARE pathway showing an age-specific decline [50,51]. In addition, oxidative stress accelerates testicular interstitial cell apoptosis and decreases testosterone synthesis. The binding of Ang (1–7) to Mas R in the RAS can exert an anti-oxidative stress effect through the sequential activation of Nrf2/HO-1 [52], and Ang II can reduce the stability of Nrf2 by prompting it to interact with Keap1, thereby attenuating its activity [53]. Therefore, long-term exogenous NT intervention may protect the function of testicular cells and maintain testosterone levels by affecting local RAS levels and activating Nrf2 to reduce oxidative stress levels. And, according to the results of the present study, it is also suggested that a long-term lack of NT intake may inhibit antioxidant pathways and increase oxidative stress levels.

In addition, we explored the effect of the biological clock regulatory gene Bmal1 in the regulation of testosterone production by exogenous NTs in this study. Some studies have revealed that testicular interstitial cells secrete testosterone according to biological rhythms, as the biological clock gene Bmal1 also regulates the expression of genes related to the synthesis and secretion of testosterone [54,55,56,57]. It has been shown that male mice with knockout of the biological clock gene Bmal1 are infertile, have reduced expression of testicular steroid genes, and have low serum testosterone levels [54,58]. Furthermore, it has been revealed that the core molecular clock protein BMAL1 controls the mRNA expression of Nrf2 to regulate its activity through direct E-box binding to its promoter [59]. And, there is evidence that the expression of major members of the RAS is regulated by circadian rhythms, and that Bmal1 silencing reduces ACE2 expression [30]. The results of the present study suggest that a chronic lack of NT intake may down-regulate the expression of the biological clock-regulated gene Bmal1, and that exogenous NTs may up-regulate Bmal1 protein expression if supplemented chronically. However, since a chronic lack of NT intake only resulted in a declining trend in testicular tissue testosterone but not a significant difference, the results have not been able to clarify the role of the biological clock gene Bmal1 in the regulation of testosterone production by NTs. Meanwhile, our study indicates that the Bmal1 gene may be an important target for exogenous NTs in exerting anti-aging effects, which will be very meaningful for future research.

Maintaining testosterone levels is important for aging men. However, the clinical benefits and long-term safety of TRT have not been fully established, and testosterone therapy is not currently FDA-approved for this indication. The long-term effects of TRT on the risk of major cardiovascular events such as myocardial infarction, stroke, and prostate cancer in aging men have been inconsistent in the findings of several clinical trials [9]. As a natural bioactive substance with high levels of safety, exogenous NTs are now widely used in powdered infant formulas [60]. Moreover, they have strong antioxidant activity and have been shown to reduce oxidative stress and activate the Sirt-1 pathway in mouse brown adipose tissue [33]. The results of the present study suggest that exogenous NTs may protect testicular cells by regulating the degree of RAS activation, activating Nrf2 and improving antioxidant capacity, thus slowing down the rate of testosterone decline in senescent SAMP8 mice, in addition to the regulation of the biological clock, which may also play a role.

However, the regulatory mechanisms of the local RAS, the role of oxidative stress, and the possible effects of the biological clock on testosterone production during aging are complex and may act synergistically with the systemic RAS [12]. The conclusions of this study on the regulatory mechanisms are suggestive, and the specific molecular mechanisms and biological processes may require more in-depth studies to explain and validate; the specific regulatory mechanisms can be further clarified by linking to kidney, in vitro, or knockout experiments. In addition, nucleotide concentrations in live mice were not measured in this study. It would be beneficial to investigate potential changes in these concentrations in a follow-up study, to determine if they are linked to the dosage of NTs administered. Also, serum testosterone levels were not tested, due to the focus on intra-organ effects in this study. The systemic effects of NTs on circulating testosterone could be explored further in future studies. Moreover, this study demonstrated the anti-testicular aging effect of NTs at the animal level, which can be further verified by conducting population experiments. Furthermore, this experiment only explored the effects of NTs on testosterone levels in male mice; however, it has been shown that the local RAS also regulates female reproductive functions [61,62], and the effects on female ovaries could be explored next, which could help to explore the effects of exogenous NTs on the reproductive system more comprehensively.

## 5. Conclusions

In summary, our results suggest that the long-term intake of exogenous NTs may delay the age-associated decline in testosterone levels in SAMP8 male mice, by modulating the RAS antioxidant pathway. The study also indicates that the biological clock may also be an important target for exogenous NTs to exert anti-organ aging effects. Our study provides a scientific basis for the functional development and mechanism of action of NTs, as well as a possible nutritional intervention program to improve testicular function.

## Figures and Tables

**Table 1:** Animals and treatments.

Groups	Sample Size	Survival Animal Numbers	Feed
NTs-F	15	13	Purified feed (0 g/kg NTs)
Control	15	12	Standard feed (1.486 g/kg NTs)
NTs-L	15	14	Standard feed + 0.3 g/kg NTs
NTs-M	15	13	Standard feed + 0.6 g/kg NTs
NTs-H	15	12	Standard feed + 1.2 g/kg NTs
SAMR1	15	12	Standard feed (1.486 g/kg NTs)

**Table 2:** Effects of exogenous NTs on oxidative stress in testicular tissue *.

	SOD (U/mg Prot)	GSH-Px (U/mg Prot)	MDA (nmol/mg Prot)
NTs-F	688.24 ± 86.88 ^c^	2.77 ± 0.77 ^c^	0.63 ± 0.25 ^c^
Control	669.39 ± 93.46 ^c^	2.91 ± 0.78 ^c^	0.78 ± 0.32 ^c^
NTs-L	870.89 ± 172.40 ^a,b^	3.64 ± 0.86 ^a,b^	0.57 ± 0.27 ^b^
NTs-M	775.08 ± 80.43 ^a,b^	3.16 ± 0.61 ^c,f^	0.51 ± 0.17 ^b^
NTs-H	795.18 ± 98.43 ^b^	3.90 ± 0.63 ^a,b,e^	0.40 ± 0.13 ^a,b^
SAMR1	816.40 ± 85.04 ^a,b^	4.00 ± 0.59 ^a,b^	0.40 ± 0.11 ^a,b^

* We randomly chose 10 mice to be tested. ^a^ Compared with the NTs-F group (*p* < 0.05); ^b^ compared with the control group (*p* < 0.05); ^c^ compared with the SAMR1 group (*p* < 0.05). Among the three NT intervention groups: ^d^ compared with the NTs-L group (*p* < 0.05); ^e^ compared with the NTs-M group; ^f^ compared with the NTs-H group (*p* < 0.05).

## Data Availability

The data presented in this study are available on request from the corresponding author. The data are not publicly available due to privacy.

## Abbreviations

SAMP8	Senescence-accelerated mouse prone-8
SAMR1	Senescence-accelerated mouse resistant 1
RAS	Renin–angiotensin system
NTs	Nucleotides
NTs-F	NTs free group
Control	Normal control group
NTs-L	Low-dose NTs group
NTs-M	Middle-dose NTs group
NTs-H	High-dose NTs group
Ang II	Angiotensin II
ACE	Angiotensin-converting enzyme
ALD	Aldosterone
PRR	Renin receptor
MR	Mineralocorticoid receptor
TRT	Testosterone replacement therapy
